# Canine leishmaniasis: the key points for qPCR result interpretation

**DOI:** 10.1186/1756-3305-4-57

**Published:** 2011-04-13

**Authors:** Verónica Martínez, Javier Quilez, Armand Sanchez, Xavier Roura, Olga Francino, Laura Altet

**Affiliations:** 1 Departament de Ciència Animal i dels Aliments, Facultat de Veterinària, Universitat Autònoma de Barcelona (UAB), 08193 Bellaterra, Barcelona, Spain; 2 Servei Veterinari de Genètica Molecular, Departament de Ciència Animal i dels Aliments, Facultat de Veterinària, Universitat Autònoma de Barcelona, 08193 Bellaterra, Barcelona, Spain; 3 Hospital Clínic Veterinari, Universitat Autònoma de Barcelona, 08193 Bellaterra, Barcelona, Spain

## Abstract

**Background:**

Diagnosis and follow up of CanL is difficult since the range of clinical signs is varied and seroprevalence is high in endemic areas. The aims of this study were: i) demonstrate the advantages of *Leishmania *qPCR to diagnose and control CanL and highlight its prognostic value and ii) propose guidelines for tissue selection and infection monitoring.

**Findings:**

This study included 710 dogs living in an endemic area of leishmaniasis. Forty percent (285/710) exhibited clinical signs consistent with CanL. Infection was detected in 36.3% (258/710) of the dogs of which 4.5% (32/710) were detected by qPCR, 16.2% (115/710) detected by ELISA and 15.6% (111/710) tested positive for both tests. Only 17.9% (127/710) of the dogs were classified sick (affected) with CanL.

All symptomatic dogs with medium or high ELISA titers were qPCR-positive in blood samples. All dogs with inconclusive or low ELISA results with high or medium qPCR parasitemia values developed the disease. Seventy one percent of asymptomatic ELISA-positive dogs confirmed by qPCR (medium to high parasitemia) developed the disease.

Bone marrow or lymph node aspirate should be selected to ensure the absence of the parasite in asymptomatic dogs: 100-1,000 parasites/ml in bone marrow are detectable in blood, whereas lower parasite loads are usually negative. Almost 10% of negative samples in blood were positive in conjunctival swabs.

**Conclusions:**

Because qPCR allows parasite quantification, it is an effective tool to confirm a diagnosis of CanL in (i) cases of inconclusive ELISA results, (ii) when the dog has not yet seroconverted, or (iii) for treatment monitoring.

## Findings

Leishmaniasis is one of the main zoonosis worldwide and in some countries it is a reason of concern for public health. Canine leishmaniasis (CanL) is of great importance in veterinary medicine since dogs are believed to be the main reservoir of this parasite for humans [1]. It is endemic along the Mediterranean basin, parts of east Africa, India, Central and South America and the incidence of infection is currently spreading to non endemic areas towards the north of Europe [2] and recently emerging in North America [3]. In addition, other species have come to be infected, such as cats [4], and horses [5]. Wild canids are competent reservoirs of *Leishmania *[6], increasing the risks for humans to acquire the disease in endemic areas. Therefore, there has been a great interest in the development of new diagnostic tests.

Diagnosis of CanL is fairly difficult, since dogs manifest a very varied range of clinical signs. In CanL, infection does not equal to having the clinical disease due to a high prevalence of subclinical infections [7,8]. Moreover, it is specially challenging in endemic areas where seroprevalence rates are high [7]. Epidemiological studies in endemic zones of CanL, by means of molecular techniques, have shown that the prevalence of infection in the canine population by *Leishmania *is considerably higher than seroprevalence [8]. There are several diagnostic tests for CanL, but the correct interpretations of these are of great importance to make an accurate diagnosis of the disease [9]. Therefore, the aims of this study were: i) to demonstrate the advantages of the quantitative PCR of *Leishmania *(qPCR) to diagnose and control the disease and highlight its prognostic value and ii) propose a guideline for the tissue of choice to be analyzed in each case, as well as a guideline for monitoring the disease.

The study included 710 dogs from the LUPA Project (7 PM; subWP canine leishmaniasis). The LUPA project http://www.eurolupa.org/ is a European initiative to study common complex human diseases using the dog as animal model. The UAB leads the subworkpackage focusing on canine leishmaniasis. All samples had informed owner consent. Data from medical history including physical exploration, clinical biochemistry and complete blood count were collected for most of the cases. Both anti-*Leishmania *ELISA and qPCR were performed on serum and blood samples, respectively. Serologies were performed at UNIVET^® ^using serum samples that were tested using INGEZIM *Leishmania*, an enzyme-linked immunoassay (ELISA) provided by Ingenasa (Madrid, Spain), with some modifications. Presence of antibodies against *Leishmania *was determined using anti-dog IgG as conjugate, following the recommendations of the manufacturer as described elsewhere [10]. qPCR was performed at the Servei Veterinari de Genètica Molecular of Universitat Autònoma de Barcelona as described by Francino *et al. *[11]. Table 1 offers the cutoff values for both ELISA and qPCR tests.

**Table 1:** Categories for cutoff values of qPCR and ELISA

qPCR result	Parasites/ml of blood	Parasites/ml of bone marrow	ELISA result	Titer (%)
**Negative**	0	0	Negative	< 20

**Low positive**	0-10	0-100	Uncertain*	20-35

**Medium positive**	10-100	100-1,000	Low positive	35-80

**High positive**	100-1,000	1,000-10,000	Medium positive	80-150

**Very high positive**	> 1,000	> 10,000	High positive	> 150

* This category was incorporated for the purpose of this study based on previous data (data not shown)

Dogs were monitorized from February 2007 until March 2010 and, according to the recorded data mentioned above, they were classified as affected or unaffected by the disease.

In 112 out of 710 dogs qPCR was additionally performed in at least one alternative tissue to detect infection of *Leishmania *(*i.e. *bone marrow, lymph node aspirate, biopsy and lesional or conjunctival swabs), providing useful information to support the tissue of choice for diagnosing CanL.

All data was compiled with Excel (Microsoft). The difference between groups was tested for significance by Chi-squared analysis. A p value < 0.05 was considered as statistically significant. For the observed prevalence, 95% confidence interval (CI) was calculated. Differences between proportions were calculated using Chi-squared Yates correction. Test characteristics such as sensitivity, disease prevalence as well as positive predictive power, were analyzed from a 2 × 2 table using ROC curve analysis. All data was analyzed using MedCalc Software [12,13].

### qPCR prognostic value

Forty percent (285/710) of the dogs exhibited clinical signs consistent with CanL. Overall detection of the parasite by ELISA and/or qPCR was found in 36.3% (258/710) of the samples, but only 17.9% (127/710) of the dogs were classified as affected of the disease. These results differ from higher values of prevalence showed in other studies where more tissues were analyzed in each dog [7], but reinforce the statement that further specific diagnostic tests such as serology and qPCR should be performed in order to reach a correct diagnosis. Treatment of dogs just based on few compatible clinical signs is not recommended, especially in endemic areas.

In endemic areas, a large part of the canine population is exposed to the parasite (positive serological results are expected) compared with the smaller proportion of dogs that eventually develop disease [7]. These results are also confirmed in our study; in which seroprevalence was of 31.8% (226/710) but only 50% of these dogs (113/226) were classified as affected of CanL. Serology titers remain high for a longer period of time before antibodies levels decrease [14]. Moreover, false positive results in serology tests can occur due to cross reactivity with other pathogens [8]. On the other hand, dogs remain seronegative during variable time periods after the infection with *Leishmania *[15]. Intervals for seroconversion on naturally infected dogs can take from 1 to 22 months (median 5 months), and from 1 to 6 months (median, 3 months) for experimentally infected dogs [16]. Therefore, serology alone has a limited predictive value because the results may be affected by persistent antibodies or inmunosupression. This statement correlates with our results because we found that 84.3% (97/115) of the dogs, who had positive serologies and negative qPCRs, were classified as unaffected of CanL. Detection and accurate parasite quantification of the qPCR is an effective help in the CanL diagnosis, mostly in cases of uncertain serology results or when the dog has still not seroconverted. Prevalence according to qPCR was lower than seroprevalence in our study (20.1% (143/710) but the majority of the dogs were classified as affected of the disease [67.8% (97/143)]. In conclusion, qPCR has a higher positive predictive value (67.8% vs. 50%; p value: 0.001) that will give us confidence when looking at test results.

**Figure 1:**
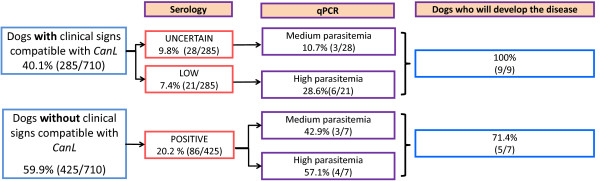
**Prognostic value of the qPCR in uncertain cases of canine leishmaniasis**.

All the dogs analyzed with clinical signs and medium or high positive ELISA titers were qPCR positive in blood samples, except those that received previous treatment or only manifested dermatological signs, in which the tissue of choice should be a swab of the dermatological lesion. In dogs with clinical signs, but low or uncertain ELISA titers, the qPCR has a significant prognostic value. In our study, 100% of symptomatic dogs with inconclusive ELISA results (uncertain or low positive), but with patent parasite detection (high or medium parasitemia measured with qPCR) develop the disease (Figure 1).

In dogs without clinical signs, the qPCR also has an important prognostic value since 71.4% of asymptomatic ELISA-positive dogs confirmed by qPCR with medium to high parasitemia values will end up with patent leishmaniasis.

As a conclusion, dogs whose parasitemia range from medium to high or very high positive are sick or eventually will become sick with CanL.

### The tissue of choice

Due to the varied tropism that the parasite exhibits regarding different tissues, the quantity of the parasite is different and oscillates among them [7]. It is important that the clinician chooses the most informative one in each case since qPCR can be performed on different tissues such as blood, bone marrow, lymph node, body fluids, histopathology samples or conjunctival swabs.

To ensure the absence of the parasite in dogs without clinical signs, the sample of choice is bone marrow or lymph node aspirate since it is been proved to be the most sensitive ones [17]. Moreover, blood is a valid tissue to perform routine qPCR analysis of CanL to evaluate response to the treatment. In our study, we found that in few samples in which both blood and bone marrow were analyzed, 77.8% (7/9) of the blood samples detected *Leishmania *while 88.9% (8/9) of the bone marrow did. The difference corresponds to two dogs that were asymptomatic and had very low titers in bone marrow (2 parasites/ml) and negative titers in peripheral blood. Parasite loads equal or greater than 100-1,000 parasites/ml in bone marrow are detectable in blood, whereas lower parasite loads (1-100 parasites/ml in bone marrow) are usually negative in blood, since there is a correlation between both tissues (bone marrow usually being higher than blood). In this, way blood is a valid tissue for monitoring treatment efficacy despite that the first qPCR diagnosis has been performed in bone marrow.

Almost 10% (8/83) of samples which were negative in blood were positive in conjunctival swab. This result correlates with other study that reported that 83% of the dogs experimentally infected with *L. infantum *were already positive by PCR of conjunctival swabs at 6 weeks after infection, whereas only 17% of the buffy coat samples obtained at the same time were found to be positive [15]. Prevalence values increase if the dog shows ocular signs such as conjunctivitis, uveitis, blepharitis or periocular alopecia. The prevalence of ocular lesions in dogs with leishmaniasis range from 16% to 80% according to various studies [18,19]. Therefore, the tissue of choice in cases of ocular signs is conjunctival swab. It could also be used for early diagnosis since sensitivity is superior to serologic testing or parasite culture [15] and samples are obtained in a less invasive manner.

**Figure 2:**
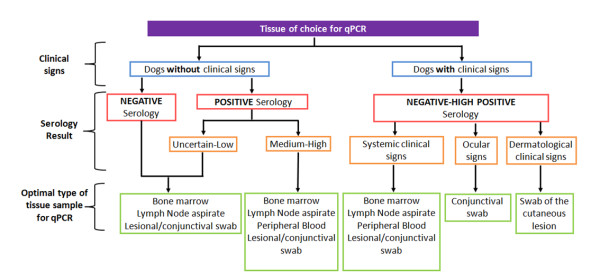
**Tissue of choice for qPCR**.

In the same way, lesional swab is recommended when only dermatological lesions are present. Other studies report that 65% of the dogs were found to be PCR positive for skin lesions compatible with *Leishmania *[15]. All together, we suggest guidelines for the tissue of choice for qPCR given prior clinical signs and ELISA results (Figure 2).

### Monitoring CanL

In cases of positive qPCR it is recommended to monitor the parasite load one month after treatment to evaluate its response. If parasite titer decreases, treatment is effective and a new qPCR control is just recommended at the end of treatment (6-12 months). None of the current anti-leishmanial drugs that are being used have proved to induce parasitological remission in dogs since a small parasite load usually remains in the majority of cases [20,21]. If the parasite load still remains positive, even after treatment, the clinician should evaluate a different treatment for the disease or the possible presence of co-infections or other diseases [22]. qPCR is an effective tool to monitor treatment efficacy especially in those cases were repeated treatments are needed and to detect early possible relapses to avoid clinical disease.

As a preventive measure, annual screening is recommended for all dogs, especially those living in endemic areas.

## Conclusions

In conclusion, due to the fact that qPCR allows quantification of parasite load in a precise manner, it could be an effective tool to confirm a diagnosis of canine leishmaniasis mostly (i) in those cases were serology is inconclusive, (ii) in cases where the dog has not yet seroconverted, (iii) for treatment monitoring. Dogs whose parasitemia range from medium to high or very high positive are sick or eventually will become sick of CanL.

## Competing interests

The authors declare that they have no competing interests.

## Authors' contributions

VMD: Redacted the manuscript. VMD and JQO: collected samples, extracted DNA, performed qPCR, performed phenotypic classification and edited the manuscript.

XR, OF, ASB and LA: edited the manuscript. All authors read and approved the final manuscript.
